# The genetic causes of male infertility: a Middle East and North Africa perspective

**DOI:** 10.12688/f1000research.106950.2

**Published:** 2022-06-06

**Authors:** Ruthwik Duvuru, Mouhammad Halabi, Temidayo S. Omolaoye, Stefan S. Du Plessis

**Affiliations:** 1 College of Medicine, Mohammed Bin Rashid University of Medicine and Health Sciences, Dubai Health Care City, Dubai, 505055, United Arab Emirates; 2 School of Medicine, Royal College of Surgeons, Ireland-Bahrain, Busaiteen, Bahrain; 3 Division of Medical Physiology, Faculty of Medicine and Health Sciences, Stellenbosch University, Cape Town, Western Cape, 7505, South Africa

**Keywords:** male infertility, chromosomal abnormalities, MENA, gene deletion, gene mutation; Y chromosome microdeletion.

## Abstract

Male infertility is attributable to *60*% of total infertility cases and about *30-50%* of these cases remain idiopathic. In the Middle East and North Africa region (MENA), male infertility affects about 22.6% of men of reproductive age. Male infertility is caused by a variety of factors, including endocrine disruption, exposure to toxins, lifestyle, genetic and epigenetic modifications. Genetic modifications, including chromosomal abnormalities, chromosomal rearrangements, Y chromosome microdeletions and single-gene mutations, explain for about 10-15% of infertility cases. Since genetic aberration is a key player in the pathogenesis of male infertility, it is important to explore the impact in the MENA region due to the high incidence of male infertility. Therefore, the current study aims to systematically analyse the literature regarding the impact and common causes of male infertility in the MENA region. To achieve this aim, a comprehensive literature search was performed on PubMed, Google Scholar, and Science Direct databases. Following the search, a total of 126 articles was retrieved, of which 12 were duplicates and another 69 articles did not meet the inclusion criteria, totaling the exclusion of 81 articles. Studies excluded were those that had patient populations originating outside the MENA region, review articles, non-English written articles, or studies where the patient population was under 18 years of age.

Findings showed that the frequent genetic aberration leading to male infertility in these regions include Y chromosome microdeletions, gene polymorphisms or copy number variations, mitochondrial microdeletions and other genetic deletions or mutations. In lieu of this, diverse clinical genetic tests should be made available for the proper diagnosis of male infertility.

## Introduction

Infertility represents the inability to achieve pregnancy after twelve or more months of regular unprotected sexual intercourse, and it affects about 15% of couples of reproductive age. Of the total cases, 50% are attributable to the male factor ( Vander Borght and Wyns 2018). It has been reported that around 60% of the total cases are attributable to the male factor, of which up to 50% are idiopathic ( Agarwal *et al.* 2019, 2021). Unlike unexplained male infertility which sometimes is characterized with normal semen parameters, idiopathic male infertility is diagnosed in the presence of altered semen characteristics without an identifiable cause and the absence of female factor infertility ( Hamada *et al.* 2012, Agarwal *et al.* 2019). Not until recently, infertility represented a reproductive health disorder that was neglected, especially in the MENA region. In 2012, Mascarenhas *et al.* reported that infertility prevalence was highest in South Asia, Sub-Saharan Africa, North Africa and the Middle East, Central/Eastern Europe and Central Asia ( Mascarenhas *et al.* 2012). Six years later, Eldib and Tashan (2018) showed that the incidence of primary infertility (inability to conceive after 12 or more months of regular unprotected sexual intercourse) in the Middle East and North Africa region (MENA) region is estimated at 3.8%, and secondary infertility (incapacity to conceive after 5 years of previous live birth) at 17.2%, while demographic infertility (failure to achieve conception with live birth within 5 years of exposure, based on a consistent union status, lack of contraceptive use, non-lactating and maintaining a desire for a child ( Mascarenhas *et al.* 2012)) is estimated at 22.6% ( Eldib and Tashani 2018). Recently, Sun *et al.* reported that the global age-standardized prevalence of infertility has increased by 23.184%, with the prevalence of male infertility estimated at 8.224%. The variations in the prevalence of male infertility across different populations were also noted ( Sun *et al.* 2019). The Western Sub-Saharan African population have the highest rates of age-standardized male infertility at 1800 infertile men per 100,000, whereas Australasia has the lowest rates, approximately 200 infertile men per 100,000 ( Sun *et al.* 2019). According to the same study, infertility rates in the MENA region are well above Central Europe, Western Europe, South-East Asia amongst several others at 800 infertile men per 100,000. Out of the three countries that presented with an increase in the trend of male infertility, two are from the MENA region. One is from the Middle East (Turkey; 1.498%) and the other is from North Africa (1.676%) ( Sun *et al.* 2019). Since demographic infertility in the MENA region is on the high side ( Eldib and Tashani 2018), and as well as the trend in male infertility ( Sun *et al.* 2019), it is of utmost importance to investigate the causes.

Utilizing the World Health Organization diagnostic classification for male infertility ( Organization 2018), studies have elucidated azoospermia, oligozoospermia, asthenozoospermia, teratozoospermia, or combinations thereof, as part of the causes of male infertility ( Ikechebelu *et al.* 2003, Punab *et al.* 2017). A study conducted in Turkey revealed that 32% of the infertility cases was due to the male factor, who were either azoospermic or oligozoospermic ( Karabulut *et al.* 2018). Even with the discovery of different causes of male infertility using semen analysis, diagnosing male infertility is complex due to a wide variety of genetic aberrations associated with the condition.

During the past decade, genetic studies have made great progress in elucidating the causes of male infertility, which include chromosomal translocations, azoospermia factor (AZF) deletions, Klinefelter syndrome, cystic fibrosis, and Noonan syndrome ( Elsawi *et al.* 1994, Okada *et al.* 1999, Sokol and Shapiro 2001, Dhanoa *et al.* 2016, Kuroda *et al.* 2020). Some studies have identified chromosomal translocations as the most common structural genetic aberration seen in men, with nearly 1.23 per 1000 ( Chen 2007, Kuroda *et al.* 2020). Until recently, genetic testing for chromosomal aberrations and AZF deletions are the only ways to come to a conclusive diagnosis of genetic abnormality induced male infertility. The optimal treatment plans for treating idiopathic male infertility have remained unclear unlike for established conditions such as hypogonadotropic hypogonadism and retrograde ejaculation. In order to get more informed about the genetic causes of male infertility, especially in the MENA region, the current study aimed to analyse the literature extensively regarding the effect, and the common genetic aberrations leading to male infertility from the MENA region perspectives. The epidemiological relevance of genetic anomalies induced male infertility was also discussed.

## Literature search

To explore the common genetic aberrations in the MENA region, a thorough literature search was performed following the methodology of the Preferred Reporting Items for Systematic reviews and Meta-Analysis (PRISMA) guidelines. Since the MENA countries include Algeria, Bahrain, Egypt, Iran, Iraq, Israel, Jordan, Kuwait, Lebanon, Libya, Morocco, Oman, Palestine, Qatar, Saudi Arabia, Syria, Tunisia, Turkey, United Arab Emirates, and Yemen, the search terms integrated each country with other parameters, such as “male infertility”, and “genetic alteration”. The literature search was performed on PubMed, Google Scholar, and Science Direct databases, retrieving articles that included male patients above the age of 18 from the MENA region, and research articles published between 1999 and 2020.

**Figure 1.**
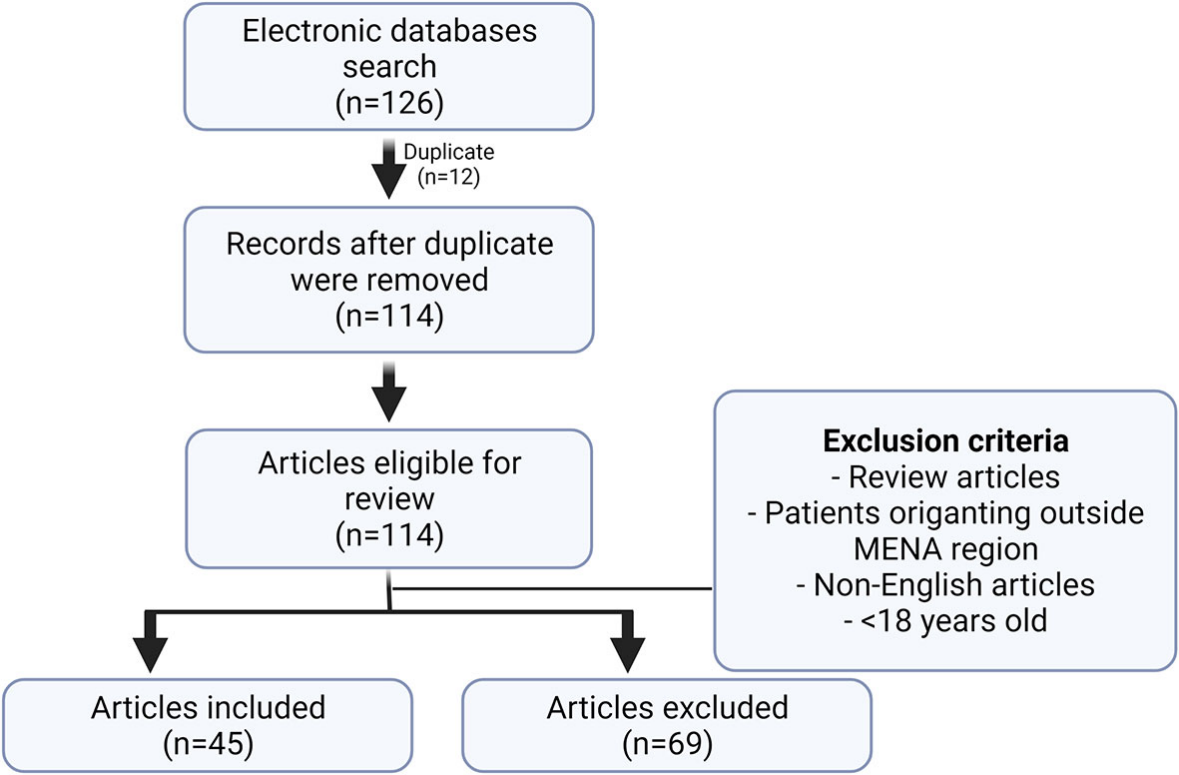
Schematic representation of the search method. Following the search from different databases, a total of 126 articles was retrieved, of which 12 were duplicates and another 69 articles did not meet the inclusion criteria. Studies excluded were those that had patient populations originating outside the MENA region, review articles, non-English written articles, or studies where the patient population was under 18 years of age.

Following the search, a total of 126 articles was retrieved, of which 12 were duplicates and another 69 articles did not meet the inclusion criteria. Studies excluded were those that had patient populations originating outside the MENA region, review articles, non-English written articles, or studies where the patient population was under 18 years of age ( Figure 1).

**Figure 2.**
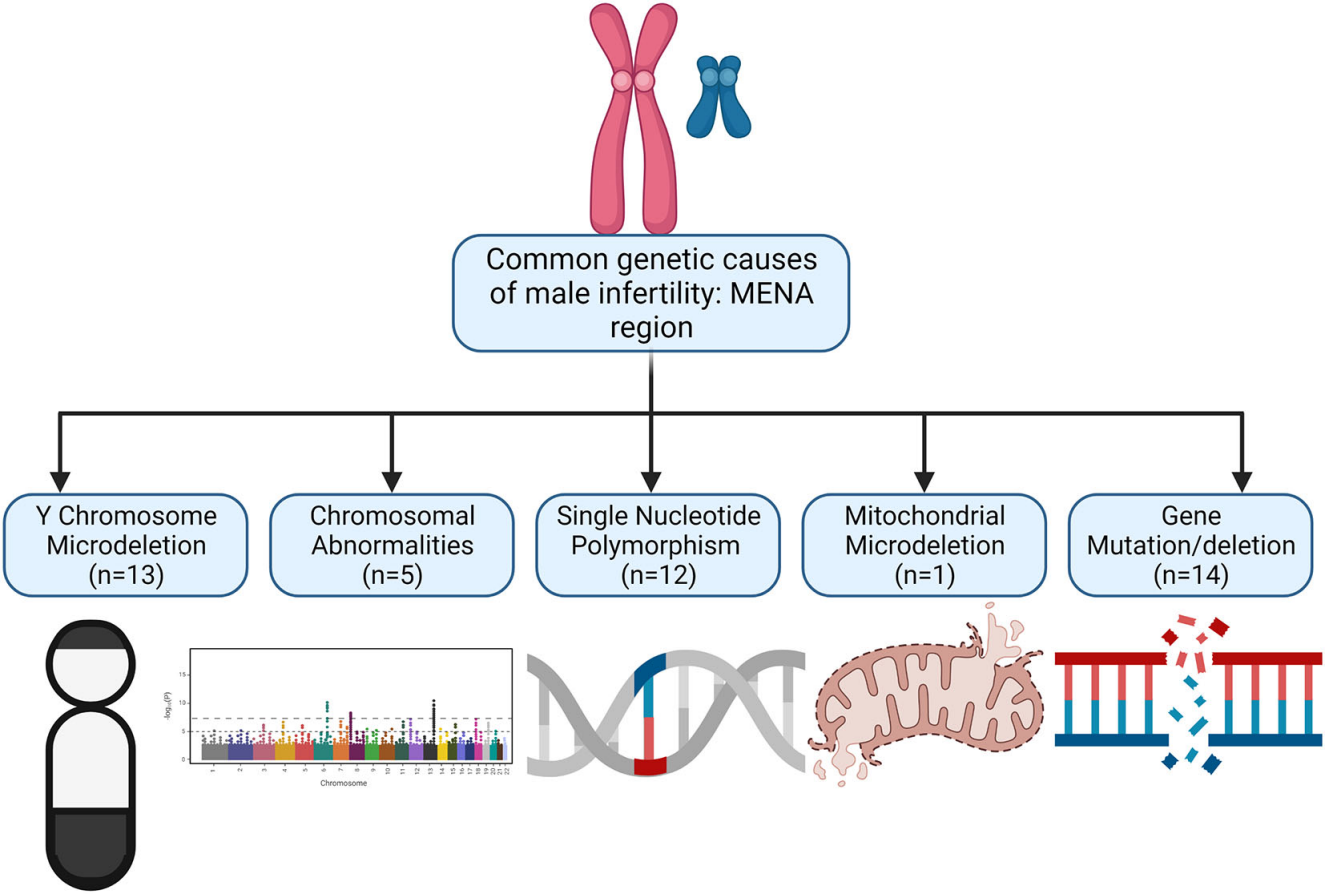
Distribution of MENA studies according to the genetic alteration. From our findings, the following are the common genetic abnormalities found in the MENA region: (i) Y chromosome microdeletion, (ii) deletion or gene mutation, (iii) gene polymorphism or copy number variations, (iv) chromosomal disorders, and (v) mitochondrial mutation.

Forty-five studies met the inclusion criteria and are reported in the current study ( Table 1). After analysing the 45 studies, 24 were performed in Iran, 14 in Turkey, 4 in Saudi Arabia, 2 from Tunisia and 1 in Iraq. Represented in Figure 2 is the distribution of MENA studies according to the genetic abnormalities. From our findings, the following are the common genetic abnormalities found in the MENA region: (i) Y chromosome microdeletion, (ii) deletion or gene mutation, (iii) gene polymorphism or copy number variations, (iv) chromosomal disorders, and (v) mitochondrial mutation. The findings will be discussed under these headings.

**Table 1.** List of studies included.

References	Country	Population phenotype	Study type	Sample/population size	Gene abnormalities	Findings
( Al-Agha *et al.* 2018)	Saudi Arabia	Azoospermia	Case study	1	Homozygous stop gain mutation in exon 6 of PSMC3IP and Missense variant in exon 1 of CLPP	Mutation of *PSMC3IP may result in infertility*
( Abur *et al.* 2019)	Turkey	Azoospermia	Case study	1300	Chromosomal aberrations and AZF microdeletion	Chromosomal aberrations and AZF microdeletions were seen in patients with either non-obstructive azoospermia or severe oligozoospermia; but could achieve successful fertilisation pregnancies with the help of assisted reproductive technology.
( Akar *et al.* 2020)	Turkey	SRY-positive 46XX testicular disorder of sex development	Case study	1300	Translocation between protein kinase X (PRKX) and inverted protein kinase Y (PRKY) genes	It is suggested that one of the underlying mechanism for 46XX is Xp:Yp translocations.
( Akbari *et al.* 2019)	Iran	Asthenozoospermia	Case study	22	Deletion in the ADCY10 coding region	Mutation of ADCY10 gene may impair sperm motility as it encodes for soluble adenylyl cyclase (sAC; the predominant adenylate cyclase in sperm).
( Akbas *et al.* 2019)	Turkey	Non-obstructive azoospermia	Case-control study	204	XRCC1 Gene Polymorphisms	XRCC1 Gene Polymorphisms is not associated with non-obstructive azoospermia
( Akgul *et al.* 2009)	Turkey	Infertile men	Retrospective study	179	Cytogenic Abnormalities	A total of 21 cases (11.74%) showed chromosomal alteration. Thirteen (7.26%) were 47,XXY; three (1.68%) were pericentric inversion of chromosome 9; one (0.56%) 46,XY/45,XO; one (0.56%) 46,XY/47,XXY/48,XXXY; one (0.56%) 46,XY,t(X;1); one (0.56%) 46,XY/46,XY,del(Y)(q11.2) and one (0.56%) 46,XX.
( Akinsal *et al.* 2017)	Turkey	46 XX testicular disorder of sex development	Retrospective	10	SRY-positive 46XX	The AZFa, AZFb and AZFc regions were absent in 8 cases. In one case, AZFb and AZFc showed incomplete deletion and normal AZFa region was present.
( Alazami *et al.* 2014)	Saudi Arabia	Oligoteratozoospermia	Case study	1	homozygous truncating mutation in NPHP4	Truncation of NPHP4 caused male infertility by altering sperm quality.
( Alimohammadi *et al.* 2020)	Iran	Globozoospermia	Case control study	104	Deletion of dpy-19 like 2 (DPY19L2) gene	Homozygous deletion of DPY19L2 was identified in 35% of men with globozoospermia. Exon 7 was deleted in 4.8% of men with globozoospermia in which DPY19L2 was not deleted, and five intronic polymorphisms were detected: 1054-77T>C in intron 9, 1131+65T>C and 1131+53A>G in intron 10 and 1218+22T>C and 1218+73T>C in intron 11. The findings suggest that DPY19L2 deletion is the/a key cause of total globozoospermia and there is no association between exons 1, 5, 8-11, 19 and 21 polymorphisms of the DPY19L2 gene in the occurrence of this defect.
( Al-Janabi *et al.* 2020)	Iraq	Azoospermia	Case control study	185	Y Chromosome microdeletion of the AZF loci	The most deleted region was AZFb region, where the incidence of microdeletions was found at 33.3%, followed by AZFc region, with a frequency of 23%, while no microdeletion was detected in AZFa.
( Asadpor *et al.* 2013)	Iran	Non-obstructive azoospermia, men whose wives have experienced more than 3 spontaneous recurrent pregnancy losses	Case control study	226	Mutation of Ubiquitin Specific Protease (USP26) (the functional gene present in AZFa region)	Total frequency of mutations in men with history of idiopathic RPL and azoospermia cases were significantly higher than that of in control groups. USP26 plays an important role in male reproduction, alterations in this gene may cause male infertility
( Askari, Karamzadeh, *et al.* 2019)	Iran	Obstructive azoospermia	Case control study	478	Variant in Claudin-2 (CLDN2) gene.	Dimeric and tetrameric arrangements of Claudin-2 were not only reduced but were also significantly altered by this single residue change. The change amino acid may likely form a polymeric discontinuous strand, which may lead to the disruption of tight junctions among epithelial cells.
( Askari, Kordi-Tamandani, *et al.* 2019)	Iran	Asthenozoospermia	Case study; Case control study	5; 430	Glutamine-Fructose-6-Phosphate Transaminase 2 (GFPT2) gene mutation	Homozygous mutation of the GFPT2 p.Arg366Gln was associated with increased levels of reactive oxygen species (ROS) in spermatozoa and decreased sperm motility.
( Aydos *et al.* 2018)	Turkey	Infertile	Case-control study	200	PRM mutation	PRM1 c.-190C>A polymorphism is associated with sperm DNA fragmentation
( Avenarius *et al.* 2009)	Iran	Male infertility	Case control study	578	Insertion mutation in the *CATSPER1 gene*	Insertion mutations (c.539-540insT and c.948-949insATGGC) led to frameshifts and premature stop codons (p.Lys180LysfsX8 and p.Asp317MetfsX18). CATSPER1 is one of four members of the sperm-specific CATSPER voltage-gated calcium channel family known to be essential for normal male fertility
( Balasar *et al.* 2017)	Turkey	Azoospermia	Case study	1		Pericentric inversion of chromosome 1 46,XY, inv(1) (p22q32)
( Beyaz *et al.* 2017)	Turkey	Oligoasthenoteratozoospermia (OAT)	Case-control study	420	gr/gr, b1/b3 and b2/b3 sub-deletions	gr/gr, b1/b3 and b2/b3 sub-deletions is not associated with OAT
( Ceylan *et al.* 2009)	Turkey	Male infertility	Case control study	165	Y chromosome Microdeletion.	Various chromosomal abnormalities and deletions of the Y chromosome can cause infertility; therefore, genetic screening is important for infertile patients
( Etem *et al.* 2010)	Turkey	Oligospermia, azoospermia, familial Mediterranean fever	Case control study	284	Mediterranean Fever Gene Mutation (M680I, M694V, M694I, V726A, P369S, and A744S)	Allelic frequencies were 2.7% for M694V and 1.35% for V726A in the infertile patient and 1.8% for M694V and 1.8% for V726A in healthy subjects. The frequency of M694V mutation is higher in the infertile group.
( Gashti *et al.* 2014)	Iran	Infertile men with varicocele	Case control study	150	4977-bp mitochondrial DNA deletion	mtDNA deletion was observed in 81.66% of patients with varicocele. Varicocele may induce mtDNA deletion in spermatozoa and cause infertility.
( Ghorbian *et al.* 2012)	Iran	Men that their wives have experienced 3 or more recurrent pregnancy loss	Case control study	200	Y Chromosome Microdeletion.	Y chromosome microdeletion is not associated with recurrent pregnancy loss
( Gurkan *et al.* 2013)	Turkey	Nonobstructive azoospermic infertile males	Case control study	130	Mutation and single nucleotide polymorphisms in the synaptonemal complex protein 3 (SYCP3) gene	No mutations were detected in the 9 exons of SYCP3. A total of eleven variations were however, detected.
( Hellani *et al.* 2005)	Saudi Arabia	Teratozoospermia	Case control study	133	Yq11 microdeletions	Teratozoospermia may be related to gonadal mosaic Y chromosome microdeletions. Y chromosome microdeletions are known to impair spermatogenesis
( Hojati *et al.* 2019)	Iran	Oligozoospermia and azoospermia	Case control study	300	Mutations in KDM3A gene	The infertile men showed various single-strand conformation polymorphism (SSCP) patterns for the exons 12 and 24. The mutations found in infertile men with otherwise unexplained severe spermatogenic failure could be considered as the origin of their abnormalities
( Jamshidi *et al.* 2014)	Iran	Teratozoospermia	Case control study	100	Fatty acid binding proteins (FABPs)	No mutation was identified in the four exons, intron 3 and splice sites of *FABP9* gene. Although previous animal studies have implicated the role of this gene in morphogenesis.
( Kamaliyan *et al.* 2018)	Iran	Idiopathic non-obstructive azoospermia	Case control study	426	Mutations and polymorphisms in HIWI and TDRD genes (the genes are critical for piRNA biogenesis and function)	There was a significant difference in the mutation of HIWI in NOA. It is suggested that there is an association between genetic variation in the HIWI2 gene and idiopathic non-obstructive azoospermia in Iranian patients, while no difference was observed in TDRD gene.
( Ben khelifa *et al.* 2011)	Tunisia	Large-headed spermatozoa	Case study	2	Aurora Kinase C gene (AURKC) mutation	There was presence of heterozygous AURKC c.144delC mutation and heterozygous variant, AURKC c.436-2A>G. These findings are important as the identification of AURKC mutations in patients indicates that all spermatozoa will be chromosomally abnormal and that ICSI should not be attempted.
( Akbarzadeh Khiavi *et al.* 2020)	Iran	idiopathic non-obstructive oligo or azoospermia infertile men	Case control study	200	*AZF region microdeletions*	Microdeletions in the AZFb and AZFc regions, and a combination of AZFb+AZFc, AZFc+AZFd and AZFb+AZFc+AZFd were reported. Suggesting karyotype and molecular analysis of Y chromosome microdeletions for genetic counselling before assisted reproduction.
( Koc *et al.* 2019)	Turkey	Sertoli-cell only syndrome	Case	39	Copy number variations of HOXD9, SYCE1, COLIA1, HI9, KCNQ1 genes	CNVs of HOXD9, SYCE1, COLIA1, HI9, KCNQ1 genes is linked with Sertoli-cell only syndrome
( Madgar *et al.* 2002)	Israel	Idiopathic infertility, nonobstructive azoospermia, severe oligospermia or azoospermia	Case control study	111	Y chromosome microdeletion and impaired androgen receptor	Y chromosome microdeletion contributes to infertility. Infertile men have longer Androgen Receptor-CAG.
( Mehdi *et al.* 2012)	Tunisia	Severe teratozoospermia	Case control study	45	Occurrence of sperm Aneuploidy	There was a significantly increased frequency of 1818, XY, XX and YY disomies in sperm with severe teratozoospermia compared with normal. The rate of total diploidy was also significantly increased.
( Mirfakhraie *et al.* 2010)	Iran	Azoospermia	Case study	100	Y chromosome microdeletion	Deletion in AZFb region was the most frequent (66.67%) followed by AZFc (41.67%), AZFd (33.33%) and AZFa (8.33%).
( Mohammad-Hasani *et al.* 2019)	Iran	Male infertility	Case control study	221	Aryl hydrocarbon receptor repressor (AhRR) transversion	Polymorphism of this gene is significantly related to the risk of male infertility
( Monsef *et al.* 2018)	Iran	NOA men with idiopathic infertility	Case control study	200	Mutation of SPATA33 gene revealed five nucleotide changes	Alterations in SPATA33 gene, at least those found in this study, may not impair spermatogenesis in patients with NOA.
( Najafipour *et al.* 2016)	Iran	Nonobstructive azoospermia, oligospermia and asthenospermia	Case control study	276	Higher frequency of YBX2 polymorphism in azoospermia. Under expression of YBX2 gene.	Under expression of YBX2 gene in the blood and testis samples of azoospermic men compared to controls, oligospermia and asthenospermia.
( Nasirshalal *et al.* 2020)	Iran	Oligoasthenoteratozoospermia	Case control study	70	Variation of the *PRM1* gene at two regulatory regions; cDNA.384G>C and cDNA.42G>A	The variations in the regulatory areas of PRM1 gene, may interfere with some critical factors related to PRM1 gene expression, hence cause male infertility.
( Ocak *et al.* 2014)	Turkey	Azoospermia or severe oligozospermia	Case study	500	Structural or numerical chromosome abnormalities; Y chromosome microdeletion; AZF deletion.	Structural or numerical chromosome abnormalities; Y chromosome microdeletion; AZF deletion.
( Pashaei *et al.* 2020)	Iran	Azoospermia, hereditary spastic paraplegia.	Case study		Homozygous variant c.375-2A > G in SYCE1	SYCE1 gene encodes synaptonemal complex (SC) central element 1 protein which contributes to the formation of the synaptonemal complex during meiosis. We suggest that the mutation 375-2A > G, which affects the acceptor splice site within intron 6 of SYCE1, is the likely cause of azoospermia and subsequent infertility in the family studied.
( Saliminejad *et al.* 2012)	Iran	Azoospermia and severe oligozoospermia.	Case control study	220	Y chromosome microdeletion	No microdeletions were detected in men with severe oligozoospermia. In the azoospermic group 2/94 (2.13%) patients showed Y chromosome microdeletions. Of the 2, one patient had complete deletion of the AZFc region and the other showed complete deletion of both the AZFb and AZFc regions.
( Shahid *et al.* 2010)	Saudi Arabia	Turner Syndrome, testicular dysgenesis syndrome	Case study	2	Deletion of cytosine in HMG box resulting in frame shift mutation	Mutation of SRY protein may be associated with the development of gonadoblastoma. It is of importance to note that mosaic patients without a SRY mutation also have a risk for malignant germ cell tumors.
( Shaveisi-Zadeh *et al.* 2017)	Iran	Azoospermia	Case control study	143	TTY2 Gene Deletion (members of testis transcript Y2 (TTY2; TTY2L12A and TTY2L2A) that are Y linked multi-copy gene families, located on Yp11 and Yq11 loci respectively)	There is a significant correlation between non-obstructive azoospermia and TTY2L12A and TTY2L2A deletions (TTY2). Thus, it seems that TTY2L12A and TTY2L2A deletions can be considered as one of the genetic risk factors for non-obstructive azoospermia
( Totonchi *et al.* 2012)	Iran	Infertile men	Case study	3654	Y Chromosome Microdeletion	The study shows that microdeletions in the AZF region should be used from a diagnostic point of view
( Vicdan *et al.* 2004)	Turkey	Non-obstructive azoospermia, oligoasthenoteratozoospermia (OAT)	Case control	228	Y chromosome microdeletion	Seventeen out of 119 (14.3%) azoospermic patients and two out of 89 (2.2%) patients with OAT had Y chromosome microdeletions. The AZFc locus, mainly DAZ gene cluster was the most frequently deleted region. Other chromosomal and genetic abnormalities were also observed in the NOA and OAT patients. This means that diverse chromosomal abnormalities and deletions of Y chromosome can cause spermatogenic breakdown resulting in chromosomally derived infertility.
( Yousefi *et al.* 2015)	Iran	Idiopathic male infertility	Case control study	300	Human apurinic/apyrimidinic endonuclease 1 (ApE1) gene mutation. Two polymorphisms -656T>G and 1349T>G ApE1 are related with the susceptibility to idiopathic male infertility	There was a significant difference in genotype distributions of -656T>G ApE1 polymorphism between infertile patients and controls. Findings indicated that individuals with the variant TG genotypes had a significant increased risk of idiopathic male infertility, whereas the significant association between the 1349T>G polymorphism and idiopathic male infertility risk was not observed. Therefore, the -656T>G ApE1 polymorphism may be associated with increased risk of idiopathic male infertility.
( Haji Ebrahim Zargar *et al.* 2015)	Iran	Azoospermia and Oligozospermia	Case control study	152	Two single nucleotide polymorphisms (SNPs) in 5΄UTR and exon 1 of H2BW gene	SNP -9C>T might contribute to complete meiotic arrest in azoospermic patients and SNP 368A>G had no correlation with male infertility.

## Y chromone microdeletion

**Figure 3.**
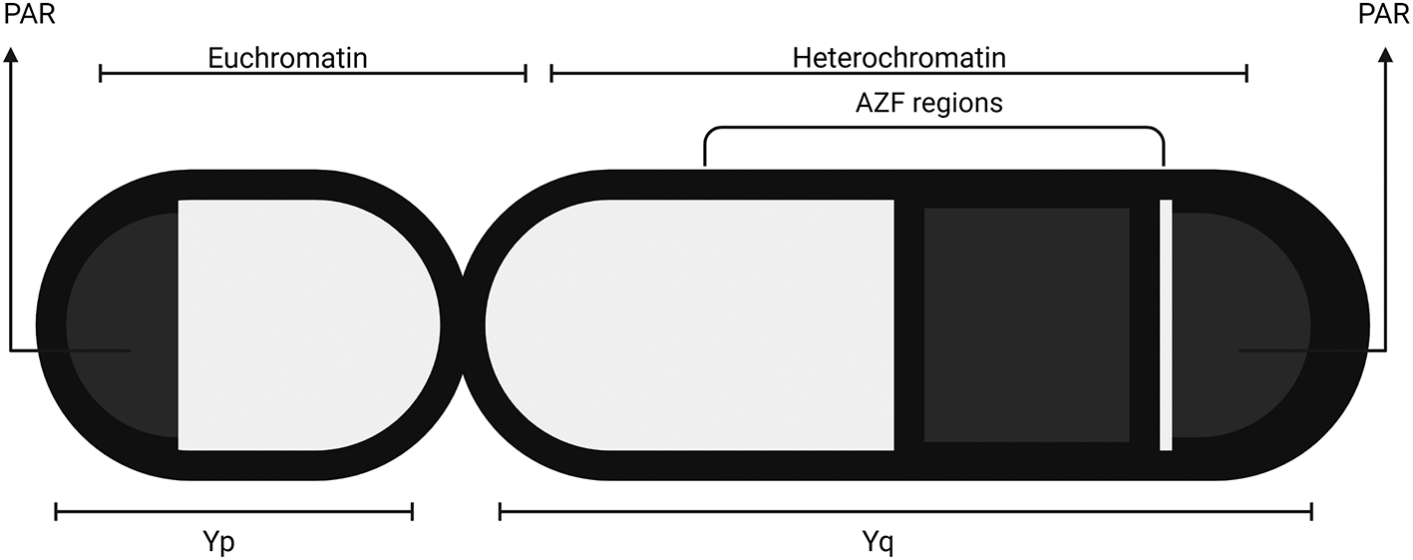
Diagram of the Y Chromosome. The Y chromosome is an acro-centric form of chromosome, with its centromere located severely off the center. It is divided into the long arm (Yq) and the short arm (Yp). The Yp and the proximal part of the Yq forms the Euchromatin, also known as the Yq11, and the distal part of Yq forms the Heterochromatin (Yq12). The pseudoautosomal region (PAR) represent where the Y chromosome binds with the X chromosome, and the AZF regions are located on the Yq. It is important to note that the length of the heterochromatin varies.

One of the most common genetic aberrations contributing to infertility is Y chromosome microdeletion. The Y chromosome is one of two sex chromosomes available within the human genome. Structurally, the Y chromosome is composed of a short arm (Yp) and a long arm (Yq) ( Ferlin *et al.* 2006, Gurkan *et al.* 2013) ( Figure 3). The long arm of the Y chromosome is made of repetitive elements that leave individuals at a high risk of internal recombination and segmental deletions. The function of the Y chromosome is to drive gonadal differentiation and develop the male phenotype ( Gurkan *et al.* 2013).

Y chromosomal microdeletions can arise in the p arm or q arm of the chromosome. If it arises in the p arm, it directly disturbs the differentiation of the testis. Y chromosome microdeletions in the AZF region of the q arm may lead to infertility. The AZF region is made up of multiple genomic loci, including AZFa, AZFb, AZFc, AZFd. These regions are believed to be responsible for spermatogenesis ( Gurkan *et al.* 2013). Variations in the AZF region may be isolated or combined. Regardless, any variation can lead to infertility.

Located in the AZFa region is Ubiquitin specific peptidase 9 Y linked ( *USP9Y*), which plays an important role in male reproductive development and spermatogenesis ( Colaco and Modi 2018), as studies have shown its absence in infertile men whilst also noting its lack even in normal sperm count fertile men ( Colaco and Modi 2018). Dead Box RNA Helicases, Box 3, Y linked ( *DBY*), another functional gene in the AZFa region, encodes an ATP-dependent DEAD-box RNA helicase that is only expressed in germ cells. It has a homologue on the X chromosome ( *DBX*) with 95% similarity, with the former playing a role limited to pre-meiotic male germ cells and the latter on post-meiotic spermatids. Males who did not have the DBY gene exhibited either Sertoli Cell only Syndrome (SCOS) or severe hypospermatogenesis, suggesting the gene’s importance in spermatogenesis ( Foresta *et al.* 2000, Stanton *et al.* 2012). The functional genes seen in AZFb include Ribosomal protein S4, Y linked *(RPS4Y2),* which is expressed in the testis and prostate ( Stahl *et al.* 2012). It plays a vital role in post-transcriptional regulation of the spermatogenic process. The Heat Shock Transcription Factor, Y linked *(HSFY),* exists as two coding copies in AZFb, *HSFY1* and *HSFY2.* *HSFY* is predominantly present in the nuclei of round spermatids and is also associated with spermatogenesis ( Stahl *et al.* 2012). One of the most important genes located in the AZFb region with 6 copies is the Ribonucleic Acid Binding Motif, Y linked ( *RBMY*) and it is responsible for the regulation of alternating splicing during spermatogenesis ( Poongothai *et al.* 2009). Deleted in Azoospermia *(DAZ)* genes are located in the AZFc region and have autosomal homologues. There are palindromic duplications of *DAZ.* These sequences together encode an RNA-binding protein vital for spermatogenesis. Infertile males with loss of DAZ seem to be highly predisposed to azoospermia and oligozoospermia ( Al-Janabi *et al.* 2020). Although, the presence of DAZ gene copies (DAZ2 or DAZ4) deletions was observed in some fertile men, the deletion of both copies were more frequent in infertile men with oligospermia ( Ghorbel *et al.* 2014). This indicate that the concurrent deletion of DAZ2 and DAZ4 gene copies is associated with male infertility, and that oligospermia seems to be promoted by deleting DAZ4 copy ( Ghorbel *et al.* 2014, Al-Janabi *et al.* 2020). Basic protein Y linked 2 *(BSY2)* is expressed in the testis and it is implicated in the process of male germ cell development. This gene is hypothesized to be involved in the cytoskeletal regulation of spermatogenesis. Testis specific protein is a multicopy gene that is only expressed in the testis and is possibly responsible for germ cell proliferation ( Ceylan *et al.* 2009, Dhanoa *et al.* 2016).

### Evidence of Y chromosome microdeletion in the MENA region

Across the different populations of the MENA region, studies have elucidated the role of Y chromosome microdeletion in male infertility ( Madgar *et al.* 2002, Vicdan *et al.* 2004, Hellani *et al.* 2005, Ceylan *et al.* 2009, Mirfakhraie *et al.* 2010, Ghorbian *et al.* 2012, Saliminejad *et al.* 2012, Totonchi *et al.* 2012, Mohammad-Hasani *et al.* 2019, Akbarzadeh Khiavi *et al.* 2020, Al-Janabi *et al.* 2020). The summary of these findings is presented in Table 1. A study carried out in Turkey by Vicdan *et al.* (2004) reported that of 208 infertile male patients, 119 had obstructive azoospermia (OA), and 89 had severe oligoasthenoteratozoospermia (OAT). Seventeen out of 119 OA patients and two out of 89 patients with OAT had Y chromosome microdeletion ( Vicdan *et al.* 2004), with the *DAZ* gene of the AZFc locus being the most frequently deleted. In total, 19 cases of Y chromosome microdeletion were detected in 208 infertile men, and chromosomal abnormalities were observed in another 5 non-obstructive azoospermia (NOA) (4.2%), and 2 OAT cases (2.2%). Of which these genetic abnormalities were not seen in the fertile men. It was also added that Y chromosome microdeletion and chromosomal abnormalities are associated with various histological alterations in the testes, such as SCOS and maturation arrest, while hypospermatogenesis occurred often in genetically normal patients.

The study conducted by Saliminejad *et al.* (2012) in Iran examined a total of 115 infertile male patients, 94 had azoospermia and 21 had severe oligozoospermia. Both patient groups were examined for Y chromosome microdeletions. Of the 94 patients with azoospermia, none had Y chromosome microdeletions, and of the 21 patients with severe oligospermia two patients were reported to have Y chromosome microdeletions. One of the patients had a deletion in the AZFc locus and the other had a deletion in the AZFb and AZFc loci ( Saliminejad *et al.* 2012). The frequency of Y chromosome microdeletion occurrence in this study is relatively low compared to other reports from the MENA region ( Vicdan *et al.* 2004, Al-Janabi *et al.* 2020).

Another study carried out in Turkey by Ceylan *et al.* (2009) reported that of the 90 infertile male patients with severe male infertility, 30 patients had NOA, 30 had oligozoospermia, and 30 were normozoospermic. Y chromosome microdeletions were present in five of the 30 patients with NOA, four of the thirty with oligospermia, and two of the normozoospermic patients. They also reported that among these patient groups the most commonly deleted Y chromosome region was the AZFc locus ( Ceylan *et al.* 2009). Chromosomal abnormalities were also seen in another 10 NOA, four oligozoospermic patients and four normozoospermic infertile men, while the 75 recruited fertile men had no deletions or chromosomal abnormalities. This shows that genetic aberration, especially Y chromosome microdeletion may be involved in idiopathic male infertility.

Hormonal aspects of Y chromosome microdeletion were reported by Mostafa *et al.* (2020) in the Iranian population. Levels of follicle-stimulating hormone (FSH) and luteinizing hormone (LH) were evaluated in fertile and infertile patients. They noted that the levels of FSH and LH were higher in Infertile men than that of their fertile counterparts, this may also serve as a reliable marker for epithelial damage, azoospermia, and Oligospermia. Additionally, high levels of testosterone and thyroid-stimulating hormone may serve as primary markers for primary testicular failure ( Akbarzadeh Khiavi *et al.* 2020).

Al-Janabi *et al*. (2020) reported that the most common region that microdeletion occurred in the sampled Iraqi population is the AZFb region, where the incidence of microdeletion was found at 33.3%. The next most common region that microdeletion occurred was the AZFc region, with a frequency of 23%. No microdeletion was reported in the AZFa region ( Al-Janabi *et al.* 2020).

Deducing from these findings, it is evident that Y chromosome microdeletion can cause several testicular dysfunctions, such as SCOS, and maturation arrest (pre- and post-meiotic), which can lead to hypospermatogenesis, NOA or OAT. Hence, the importance of testing for Y chromosome microdeletion in men experiencing idiopathic infertility should be promoted in the MENA region.

## Genetic mutations

Genes control a variety of physiological processes, including reproductive developments. Spermatogonial stem cells must undergo a variety of processes before becoming fully matured spermatozoa; these phases are controlled by genes. Any variation in genes that contribute to sperm maturation may lead to infertility.

Genetic abnormalities account for 15-30% of infertility cases worldwide ( Kovac and Alexander W. Pastuszak 2014), hence, identifying and understanding the various genetic mutations is vital. It is important to recognize the genetic basis of infertility to provide better care, as well as an improved prognosis to infertile couples. Several studies have shown how the variation in essential spermatogenesis specific genes led to the impairment of this process and ultimately male infertility ( Avenarius *et al.* 2009, Shahid *et al.* 2010, Etem *et al.* 2010, Asadpor *et al.* 2013, Jamshidi *et al.* 2014, Alazami *et al.* 2014, Shaveisi-Zadeh *et al.* 2017, Al-Agha *et al.* 2018, Monsef *et al.* 2018, Akbari *et al.* 2019, Askari, Karamzadeh, *et al.* 2019, Askari, Kordi-Tamandani, *et al.* 2019, Hojati *et al.* 2019, Alimohammadi *et al.* 2020). This section will briefly describe some genes that the deletion or mutation thereof led to impaired male fertility.

### Evidence of genetic mutations in the MENA region

*Glutamine-Fructose-6-Phosphate Transaminase 2*

A study done in Iran by Askari *et al.* (2019) discussed the effects of variation in Glutamine-Fructose-6-Phosphate Transaminase 2 ( *GFPT2*) on fertility. *GFPT2* is a rate-limiting enzyme that is responsible for hexosamine biosynthesis. They found that a homozygous missense mutation in the gene led to azoospermia. They also noted that *GFPT2* may protect against reactive oxygen species (ROS); ROS may induce the peroxidation of unsaturated fatty acids or phosphorylate axoneme proteins. Both mechanisms eventually lead to decreased sperm motility ( Askari, Kordi-Tamandani, *et al.* 2019).

*Lysine demethylase 3A pathway*

A study carried out by Hojati *et al*. (2019) examined the relationship between variation in lysine demethylase 3A ( *KDM3A*) and male Infertility. *KDM3A* is a gene that is believed to be responsible for sperm chromosome condensation. The study reported that various mutations in the *KDM3A* gene led to infertility in five Iranian males ( Hojati *et al.* 2019). To rule out the common causes of infertility, they also examined Y chromosome microdeletion and partial AZF deletions. Surprisingly, the five patients with variation in *KDM3A* had no Y chromosome microdeletion or AZF microdeletion. This study proves that a variations in *KDM3A* could lead to spermatogenic failure. They also pointed out that the *KDM3A* gene is located on chromosome 2, which can be transferred to the offspring via the genetic pool. This means that the offspring, regardless of gender, could be susceptible to inheriting this type of mutation.

*CATSPER channel protein*

Avenarius *et al*. (2009) carried out a study to report the relationship between variation in the CATSPER1 channel and infertility amongst two Iranian men. The study was able to identify insertion mutations, which led to premature stop codons and consequently variation in the CATSPER 1 protein ( Avenarius *et al.* 2009). The CATSPER1 protein is part of a tetrameric voltage gated calcium channel which are highly conserved in humans and mice. Carlson *et al.* showed the necessity of CATSPER1 for Ca2 ^+^ entry into the flagellum and for Ca2 ^+^ -mediated hyperactivated sperm motility ( Carlson *et al.* 2003). Thus, an abnormality in the CATSPER1 protein may impede the calcium-mediated sperm functions. Once sperm enters the female reproductive tract, it undergoes the calcium mediated process of capacitation. When capacitation occurs successfully, the sperm is able to carry out its role in fertilization. Thus, it was suggested that variation in the CATSPER1 channel may hinder the process of capacitation and consequently leading to infertility ( Avenarius *et al.* 2009).

*Spermatogenesis associated 33 mutation (SPATA33)*

This study performed by Monsef *et al.* (2018) examined the relationship between variations in *SPATA33* and infertility in men with NOA. *SPATA33* is highly expressed in the testis, and it is believed to be highly expressed during the first wave of spermatogenesis, indicating its possible association with the meiotic process. Therefore, it was reasonable to assume that a variation of this gene might lead to infertility. However, it was reported that there is no direct association between *SPATA33* mutation and infertility in men with NOA. The authors discuss that the study population was limited to men with NOA and encouraged that the same study be done in men with oligospermia and teratozoospermia ( Monsef *et al.* 2018).

*Piwi interacting RNA pathway*

A study carried out by Kamaliyan *et al.* (2018) investigated the PiRNAs, which are amongst the non-coding regions of RNA and male germline development. *PIWI* and *TDRD* genes are essential for PiRNAs to function appropriately, hence they are necessary for proper spermatogenesis. The study examined the association between polymorphisms in the *HIWI* genes and the risk of idiopathic non-obstructive azoospermia in Iranian males. Variations may cause RNA instability. Evidently, any variants in the PiRNA pathway genes may predispose spermatogenesis defects ( Kamaliyan *et al.* 2018).

Extrapolating from the results, it can be suggested that the mutation or deletion of genes necessary for normal development of germ cells, even without the presence of Y chromosome microdeletion may impair male fertility by triggering altered spermatogenesis, reduced sperm function, and some may even cause the offspring to be prone to inheriting the variation. Hence, it is important to identify if male infertility is caused by a gene mutation. This will help to develop treatment strategies that would prevent the offspring from having the same mutation.

*X-ray repair cross complementing group 1 genetic polymorphism*

DNA is under constant threat and damage from various sources. The X-ray Cross Complementing Group 1 ( *XRCC1*) gene is responsible for repairing single strand breaks in the DNA. Mutations in the *XRCC1* are detected by using polymerase chain reaction reaction-restriction fragment length polymorphism ( Bi *et al.* 2013). A study by Akbas *et al.* examined polymorphisms within the *XRCC1* gene and their effect on male fertility. A control group was compared to a group with men that suffered from idiopathic non-obstructive azoospermia. No significant differences were reported in *XRCC1* polymorphisms between the control and experimental group, suggesting that *XRCC1* polymorphisms do not influence male fertility ( Akbas *et al.* 2019).

*Protamine (PRM) and Y-box binding protein 2 (YBX2)*

Protamine *(PRM)* genes produce protamine, which are small arginine rich proteins and are believed to be essential for DNA stabilization and function to condense spermatid genome ( Domenjoud *et al.* 1991). Y-box binding protein 2 *(YBX2)* is essential in the transcription, translation, and splicing of mRNA. A study by Aydos *et al.* aimed to demonstrate the effects of polymorphism in such genes, and whether they can potentially affect male fertility. It was reported that *PRM1* polymorphism was associated with sperm DNA fragmentation, while a polymorphism in *PRM2* and *YBX2* were not associated with male fertility ( Aydos *et al.* 2018).

## Single nucleotide polymorphisms (SNPs)

Single nucleotide polymorphisms (SNPs) are the replacement of a nucleotide at a single position within the genome, giving rise to a new allele. A SNP may occur anywhere along the genome, affecting genetic integrity. If it occurs on the sex chromosomes it may hinder the maturation of sperm, leading to infertility ( Ben khelifa *et al.* 2011, Gurkan *et al.* 2013, Haji Ebrahim Zargar *et al.* 2015, Yousefi *et al.* 2015, Najafipour *et al.* 2016, Kamaliyan *et al.* 2018, Nasirshalal *et al.* 2020, Pashaei *et al.* 2020). Understanding the specifics of where the gene is mutated, and how it can lead to male infertility is vital in the treatment and management plan of the patient.

### Evidence of SNPs occurrence in the MENA region

A study conducted by Zargar *et al*. (2015) discussed the relationship between variation in the X-linked gene and a specific pattern of male infertility. They reported that a gene on the x chromosome, known as H2B.W, is linked to male infertility ( Haji Ebrahim Zargar *et al.* 2015). The study discovered two SNPs (-9C>T and 368A>G) in the *H2B.W* gene in a population of infertile Iranian men.

The study showed that the -9T frequency at the -9C>T position was higher in the complete maturation arrest group than in the SCOS group. This suggests that the variation of allele C to T might influence the mRNA stability affecting the maturation of the spermatids. However, there was no significant association between SNP 368A>G and the risk of infertility in the Iranian male population ( Haji Ebrahim Zargar *et al.* 2015).

Another study analysed the whole blood samples of 180 idiopathic infertile males and 120 fertile controls to investigate the association between the occurrence of gene polymorphism (-656T>G and 1349>G variants in the ApE1 promoter and coding region) and the susceptibility to idiopathic male infertility ( Yousefi *et al.* 2015). ApE1 is responsible for maintaining genomic integrity, a polymorphism in this gene might lead to infertility as it may cause damage to the DNA leading to reproductive disorders. The study revealed that -656T>G polymorphism is related to infertility, while a variation in the 1349T>G region was unrelated to idiopathic male infertility ( Yousefi *et al.* 2015).

## Chromosomal disorders

The implication of chromosomal disorders on male infertility including numerical, structural, replacement, inversion, insertion and translocational chromosomal abnormalities have been explored and documented ( Balkan *et al.* 2008, Akgul *et al.* 2009, Alhalabi *et al.* 2013), especially for the numerical and structural chromosome disorders ( Balkan *et al.* 2008, Alhalabi *et al.* 2013).

### Evidence of chromosomal disorders in the MENA region

Coming to the MENA region, Mehdi *et al.* (2012) reported a significantly increased frequency of chromosome 1818XY, XX, and YY disomies in the spermatozoa of men with severe teratozoospermia from Tunisia ( Mehdi *et al.* 2012). The rate of total diploidy was also increased. Another study from Turkey showed that out of 179 infertile men that were evaluated, a total of 21 cases (11.74%) showed chromosomal alteration. This include 13 (7.26%) that were 47,XXY; three (1.68%) were pericentric inversion of chromosome 9, one (0.56%) 46,XY/45,XO, one (0.56%) 46,XY/47,XXY/48,XXXY, one (0.56%) 46,XY,t(X;1), one (0.56%) 46,XY/46,XY,del(Y)(q11.2), and one (0.56%) 46,XX ( Akgul *et al.* 2009). The occurrence of diploidy originating from either meiotic maturation or by a compromised testicular environment may impair male fertility. A case report by Balasar *et al.* demonstrates that not all chromosomal mutations will result in variation in the AZF and SRY regions ( Balasar *et al.* 2017), which demonstrates the importance of understanding the differences in variation to properly treat infertility.

## Mitochondrial mutation

The mitochondrion is a double-membrane organelle that generates about 90% of cell energy in the form of adenosine triphosphate by oxidative phosphorylation reaction in mammalian cells. Mitochondria play a crucial role in a series of signal pathways, including tricarboxylic acid cycle, the β-oxidation of fatty acids, regulation of intrinsic apoptosis, and participating in the cell cycle ( Arakaki *et al.* 2006, Finkel and Hwang 2009, Yan *et al.* 2019). In contrast to the other organelles in a mammalian cell, mitochondria have DNA, known as mitochondrial DNA (mtDNA), which encodes a series of crucial proteins for mitochondrial respiration. The mtDNA is particularly susceptible to certain stress-induced damages due to a lack of histones in the structure and effective repair mechanisms ( Kujoth *et al.* 2005) mtDNA mutation caused by stress-induced damage is highly associated with various human diseases, including male infertility ( Venkatesh *et al.* 2009).

### Evidence of mitochondria mutation in the MENA region

Abnormal sperm function has been identified as one of the leading causes of male infertility. Defective sperm motility has been recognized as one of the primary causes of abnormal sperm function. Gashti *et al*. (2013) reported that variations in mtDNA in ATP generating genes may cause infertility, as mtDNA deletion was observed in 81.66% of infertile men with varicocele. This means that varicocele may induce mtDNA deletion in spermatozoa and cause infertility ( Gashti *et al.* 2014). They also reported that ROS in testicular tissue and semen may lead to mtDNA microdeletions, which affects the electron transport chain; which is consequently a direct cause of male infertility ( Gashti *et al.* 2014). Many factors can contribute to mtDNA damage, such as infection, lifestyle, diet, and the environment. These factors promote the production of ROS, and subsequently leads to the development of oxidative stress when sustainably increased. At a high level of oxidative stress, spermatozoa may be damaged, thus promoting male infertility. Several studies have reported the adverse role of excessive ROS on male fertility ( Ciftci *et al.* 2009, Ni *et al.* 2016). These negative effects are in-part exerted due to the susceptibility of the sperm plasma membranes which are rich in poly-unsaturated fatty acids to excessive and sustained generation of ROS ( Du Plessis *et al.* 2010, 2015). In lieu, a study investigated the implication of mtDNA damage on male fertility in a cohort of Iranian population. It was reported that infertile men displayed multiple deletions of the mtDNA, suggesting that deletions of the mtDNA may be a risk factor for male infertility ( Talebi *et al.* 2018).

## Clinical implications

In vitro fertilization (IVF) and Intracytoplasmic Sperm Injection (ICSI) have allowed couples with fertility problems to achieve success. The success of these procedures varies from couple to couple because different couples present with diverse causes of male infertility. A study by Ocak *et al.* explored the causes of reproductive failure in a cohort of 500 patients. They found that the causes of infertility ranged from no chromosomal variations to Y-chromosomal variation. Thus, demonstrating the importance of genetic testing before commencing assisted reproductive techniques (ART) ( Ocak *et al.* 2014). With that being said, most patients are still willing to attempt such procedures, as these procedures present as a last hope option.

Y chromosome microdeletion is one of the most common causes of male infertility; many males who suffer from Y chromosome microdeletion undergo IVF and ICSI. Screening for Y chromosome microdeletion has become a standard practice before partaking in either IVF or ICSI, as they may offer a prognostic value, predicting the potential success for ART ( Sadeghi-Nejad and Farrokhi 2007). Knowing the type of Y chromosome microdeletion may help offer some prognostic value, as not all types of microdeletions yield the same results with ART. It has been demonstrated that sperm retrieval through testicular sperm extraction was possible in patients with AZFc microdeletion but not possible in AZFa and AZFb ( Krausz *et al.* 2000, Hopps *et al.* 2003). A more recent study by Abur *et al.* also demonstrated that ART was possible with AZFc deletion ( Abur *et al.* 2019), marking the importance of differentiating between types of Y-chromosome microdeletion before commencing ART. Other chromosomal abnormalities may affect the success rate of ART; such an example would be 46 XX chromosomal abnormalities. Akar *et al.* reported that other than the clinical and laboratory findings of 46 XX chromosomal translocation, patients with such a condition may have to resort to a sperm donor as sperm retrieval is not a viable option in such a patient population ( Akar *et al.* 2020). Furthermore, this patient population should opt for testosterone replacement therapy to be protected against the negative effects of testosterone deficiency ( Akinsal *et al.* 2017).

Additionally, high levels of aneuploidy are positively associated with an increased level of male factor infertility ( Schulte *et al.* 2010). As such, sperm with aneuploidy is associated with a higher rate of failure with ART ( Harton and Tempest 2012). Sperm relies on energy from the mitochondria for its motility, therefore, any variation in mtDNA leads to altered motility, negatively impacting fertility outcomes. A proposed solution for such infertility is ICSI. Studies now show that although mitochondrial DNA variation has a negative impact on ICSI outcomes, it is still possible ( Al Smadi *et al.* 2021). Sperm DNA integrity is one of the vital prognostic factors of male fertility. Anything that compromises sperm DNA can lead to infertile outcomes. The findings on IVF outcomes in patients with abnormal sperm DNA have been conflicting. Some studies state that variation in the DNA of sperm have no effect on fertility outcomes ( Collins *et al.* 2008), while others state otherwise ( Simon *et al.* 2017). The controversy may be due to the diversity methodological approaches. Hence, it is suggested that a standardized protocol be developed.

Upon achieving success with ART, the main concern shifts to the possible vertical transmission to the offspring. Reports have shown that microdeletions have the capability of transmitting to the offspring by ICSI ( Jiang *et al.* 1999, Zhu *et al.* 2010). Unfortunately, vertical transmission of Y chromosome microdeletion have been reported to cause infertility in offspring ( Kim *et al.* 2003, Dai *et al.* 2012). Studies have also shown that males with aneuploidy have a higher chance of giving birth to children with aneuploidy which can translate to a variety of health conditions ( Harton and Tempest 2012). This dilemma requires the design of further prospective clinical cohort studies that will assess whether the deleted regions on the Y chromosome are amplified and whether they can cause any significant new health consequences. Investigations on the possible transmission of damaged DNA should also be developed.

## Conclusion

In comparison to the data available on the global investigation of infertility, particularly male infertility, findings about this subject in the MENA region is lacking. This may be due to the poorly funded niche-specific research, or social stigmatization. Accessibility to the few studies has revealed that the prevalence of demographic male infertility in the MENA region is on the increase, which makes the investigation of the causes of male infertility important.

In addition to semen analysis derived diagnosis, studies have indicated the role of genetic abnormalities as part of the cause of male infertility. Findings from the current study showed that the prevalent genetic aberration leading to male infertility in the MENA region include Y chromosome microdeletion, the occurrence of gene polymorphism, mitochondrial microdeletion and other genetic deletions or mutations.

The study of male infertility in the MENA region should encompass the investigation of various genetic variations. Diverse clinical genetic tests should also be made available for the proper diagnosis of male infertility. This would furthermore help researchers and clinicians to develop informed treatment strategies. Additionally, before providing couples with ART options, a thorough screening should be performed, and the scope of interest of reproductive medicine physicians should as well include understanding the root cause of infertility rather than just establishing pregnancy.

## Data availability

No data is associated with this article.
