# Supplementary material for: The genetic causes of male infertility: a Middle East and North Africa perspective
Source: F1000Res. 2022 Jun 6;11:125. Originally published 2022 Jan 31. [Version 2] doi: 10.12688/f1000research.106950.2 (PMC9641111; doi:10.12688/f1000research.106950.2)
Supplement: Peer review history

# Peer review: Reviewer response for version 2

**DOI:** 10.5256/f1000research.134493.r139754

**Published:** 2022-11-07

**Authors:** Arnold Peter Paul Achermann

**Affiliations:** 1 Department of Surgery, Division of Urology, Faculty of Medical Sciences, University of Campinas (UNICAMP), Campinas, Brazil

This manuscript has been well-reviewed and modified. All the issues questioned were analyzed, reconstructed with a better approach. Congratulations for the elaboration of this article.

Is the review written in accessible language?

Partly

Are all factual statements correct and adequately supported by citations?

Partly

Are the conclusions drawn appropriate in the context of the current research literature?

Yes

Is the topic of the review discussed comprehensively in the context of the current literature?

Yes

Reviewer Expertise:

Male infertility, micro-TESE, azoospermia, Non-obstructive azoospermia, hypogonadism

I confirm that I have read this submission and believe that I have an appropriate level of expertise to confirm that it is of an acceptable scientific standard.

# Peer review: Reviewer response for version 2

**DOI:** 10.5256/f1000research.134493.r139755

**Published:** 2022-06-13

**Authors:** Małgorzata Piasecka

**Affiliations:** 1 Department of Histology and Developmental Biology, Faculty of Health Science, Pomeranian Medical University, Szczecin, Poland

I have no objection to the revised manuscript. The authors responded to my comments and made corrections that I suggested.

Is the review written in accessible language?

Partly

Are all factual statements correct and adequately supported by citations?

Yes

Are the conclusions drawn appropriate in the context of the current research literature?

Yes

Is the topic of the review discussed comprehensively in the context of the current literature?

Yes

Reviewer Expertise:

Basic and non-conventional seminological studies, genetic aspects of male infertility, sperm biology

I confirm that I have read this submission and believe that I have an appropriate level of expertise to confirm that it is of an acceptable scientific standard.

# Peer review: Reviewer response for version 1

**DOI:** 10.5256/f1000research.117957.r135107

**Published:** 2022-05-05

**Authors:** Arnold Peter Paul Achermann

**Affiliations:** 1 Department of Surgery, Division of Urology, Faculty of Medical Sciences, University of Campinas (UNICAMP), Campinas, Brazil

In this paper, the authors summarize the current evidence concerning the importance of the genetic causes of male infertility in the Middle East and North Africa (MENA) region.

 The Abstract offers a clear, synthetic, and complete overview of the messages emerging from the Review. However, the authors should update information about the percentage of idiopathic cases in male infertility.

 Although difficult to read of the complex theme, the Review is written linearly and deals with everything related to genetic causes of male infertility in the MENA region in separate chapters.

 I suggest minor corrections:

-  "The AZF region is made up of multiple genomic loci, including AZFa, AZFb, AZFc, AZFd. These regions are believed to be responsible for spermatogenesis" The authors should add that deletions can be isolated or combined in each AZF region.
- "Albeit that the presence of DAZ deletions in both fertile and infertile men question its importance, the former although fertile have lower sperm counts and reduced sperm motility." Confused, clarify.
- Is the KDM3A gene an autosome dominant or recessive gene?
- The paragraph about (SPATA33) is about the Iranian population studied. However, the authors did not expose it.
- "Nasrin also reported that ROS in testicular tissue and semen may lead to mtDNA microdeletions, which affects the electron transport chain, which is consequently a direct cause of male infertility." – there is no reference for this information.
- "It has been demonstrated that sperm retrieval through testicular sperm extraction was possible in patients with AZFC microdeletion but not possible in AZFA and AZFB (Krausz et al. 2000, Hopps et al. 2003). A more recent study by Abur et al. also demonstrated that AR was possible with AZFC deletion (Abur et al. 2019), marking the importance of differentiating between types of Y-chromosome microdeletion before commencing ART." – please correct the expressions. It should be AZFa, AZFb, AZFc.

Is the review written in accessible language?

Partly

Are all factual statements correct and adequately supported by citations?

Partly

Are the conclusions drawn appropriate in the context of the current research literature?

Yes

Is the topic of the review discussed comprehensively in the context of the current literature?

Yes

Reviewer Expertise:

Male infertility, micro-TESE, azoospermia, Non-obstructive azoospermia, hypogonadism

I confirm that I have read this submission and believe that I have an appropriate level of expertise to confirm that it is of an acceptable scientific standard, however I have significant reservations, as outlined above.

# Response

**Published:** 2022-05-29

**Authors:** Temidayo S Omolaoye

**Affiliations:** Mohammed Bin Rashid University of Medicine and Health Sciences, United Arab Emirates

**Comment:**

 In this paper, the authors summarize the current evidence concerning the importance of the genetic causes of male infertility in the Middle East and North Africa (MENA) region. The Abstract offers a clear, synthetic, and complete overview of the messages emerging from the Review. However, the authors should update information about the percentage of idiopathic cases in male infertility.

 Although difficult to read of the complex theme, the Review is written linearly and deals with everything related to genetic causes of male infertility in the MENA region in separate chapters.

**Response:**

 The authors would like to thank the reviewer for the positive remark and for taking the time and effort to review this work, thereby adding to the scientific merit of this study. Suggestions made by the reviewer have been addressed appropriately. 

 The prevalence of idiopathic cases in male infertility have been updated both in the abstract and main text. 

**Comment:**

 I suggest minor corrections:

  "The AZF region is made up of multiple genomic loci, including AZFa, AZFb, AZFc, AZFd. These regions are believed to be responsible for spermatogenesis" The authors should add that deletions can be isolated or combined in each AZF region.

**Response:**

 The suggested comment has been added in the text. Line 139-140

**Comment:**

 "Albeit that the presence of DAZ deletions in both fertile and infertile men question its importance, the former although fertile have lower sperm counts and reduced sperm motility." Confused, clarify.

**Response:**

 The below sentence has been inserted in the text. Line 163-167

 “Although, the presence of DAZ gene copies (DAZ2 or DAZ4) deletions was observed in some fertile men, the deletion of both copies were more frequent in infertile men with oligospermia (Ghorbel *et al.* 2014). This indicate that the concurrent deletion of DAZ2 and DAZ4 gene copies is associated with male infertility, and that oligospermia seems to be promoted by deleting DAZ4 copy (Ghorbel *et al.* 2014, Al-Janabi *et al.* 2020).”

**Comment:**

 Is the KDM3A gene an autosome dominant or recessive gene?

**Response:**

 According to the available literature, the dominant or recessive status of KDM3A gene is not yet clarified. However, this gene encodes a zinc finger protein that contains a jumonji domain and plays a role in hormone-dependent transcriptional activation by participating in recruitment to androgen-receptor target genes, which result in H3 'Lys-9' demethylation and transcriptional activation. Additionally, it is involved in spermatogenesis by regulating expression of target genes such as PRM1 and TNP1 which are required for packaging and condensation of sperm chromatin. It functions suggest that it may be a dominant gene.

**Comment:**

 The paragraph about (SPATA33) is about the Iranian population studied. However, the authors did not expose it.

**Response:**

 Findings from the study on SPATA33 has been given in details in the text.

**Comment:**

 "Nasrin also reported that ROS in testicular tissue and semen may lead to mtDNA microdeletions, which affects the electron transport chain, which is consequently a direct cause of male infertility." – there is no reference for this information.

**Response:**

 The reference has been inserted.

**Comment:**

 "It has been demonstrated that sperm retrieval through testicular sperm extraction was possible in patients with AZFC microdeletion but not possible in AZFA and AZFB (Krausz et al. 2000, Hopps et al. 2003). A more recent study by Abur et al. also demonstrated that AR was possible with AZFC deletion (Abur et al. 2019), marking the importance of differentiating between types of Y-chromosome microdeletion before commencing ART." – please correct the expressions. It should be AZFa, AZFb, AZFc.

**Response:**

 Changes have been affected in the entire manuscript.

# Peer review: Reviewer response for version 1

**DOI:** 10.5256/f1000research.117957.r126419

**Published:** 2022-03-30

**Authors:** Małgorzata Piasecka

**Affiliations:** 1 Department of Histology and Developmental Biology, Faculty of Health Science, Pomeranian Medical University, Szczecin, Poland

The manuscript presented to me for review concerns the genetic causes of male infertility in the Middle East and North Africa (MENA) region. The topic discussed is important and still relevant due to its clinical significance and implications. Based on a literature review (different electronic databases were searched), the authors showed that genetic aberrations (Y chromosome microdeletion, deletion or gene mutation, gene polymorphism or copy number variation, chromosomal disorders, mitochondrial mutation) are a common cause of male infertility in the MENA region. The obtained research results are comprehensively presented in Table 1 and described in the text. Taking into account the obtained data, the authors rightly suggest that genetic tests should be part of the diagnosis of male infertility. Undoubtedly, such diagnostics will allow for proper therapeutic management, especially in the case of using ART. Generally, the text is written correctly in terms of its content. However, the manuscript requires revision prior to indexing.

 The most important remarks are listed below:

- Abstract: “…30% of these cases remain idiopathic.” This data should be updated. Idiopathic infertility accounts for 30-50% of male infertility (Agarwal et al., 2019,2021).
- There is no information in the Introduction about idiopathic failure.
- “Y chromone (?) microdeletion”; “…P or Q arm of…” should be p arm or q arm
- The content on the molecular structure of the Y chromosome should also be presented in the form of a diagram (structure of the Y chromosome with regions and genes marked).
- The caption under figure 2 should be corrected: ”…the genetic alteration the genetic alteration.”
- Information on ROS is contained in two sections: “Evidence of genetic mutations in the MENA region” and “Evidence of mitochondria mutation in the MENA region”. I suggest that they be written exhaustively only in the section: “Evidence of mitochondria mutation in the MENA region”. They should be expanded and superseded with the latest literature. The text includes many mental abbreviations.
- Section: “CATSPER channel protein”. “Avenarius et al. (2009) carried out a study to report the relationship between variation in the CATSPER1 channel and infertility **amongst **(???) Iranian men. The study showed that the CATSPER1 channel protein is responsible for the calcium influx during the hyperactivity phase of sperm after ejaculation. Thus, any variation in this protein channel can disrupt the hyperactivity phase, evidently leading to infertility (Avenarius et al. 2009).” This fragment of text should be corrected. Hyperactivation of ejaculated sperm cells (the spermatozoa movement is changed) is the result of their capacitation in the female reproductive system and enables the fertilization process. The authors used too much shortcut. References should be updated.
- No development of the abbreviation PiRNAs, TDRD;  “ *PIWI* (comment of reviewer: gene or protein,  italics means gene) and TDRD proteins are…”… *HIWI *genes… “(???)
- The text should be read very carefully and checked in terms of spelling, style and abbreviations.

Is the review written in accessible language?

Partly

Are all factual statements correct and adequately supported by citations?

Yes

Are the conclusions drawn appropriate in the context of the current research literature?

Yes

Is the topic of the review discussed comprehensively in the context of the current literature?

Yes

Reviewer Expertise:

Basic and non-conventional seminological studies, genetic aspects of male infertility, sperm biology

I confirm that I have read this submission and believe that I have an appropriate level of expertise to confirm that it is of an acceptable scientific standard, however I have significant reservations, as outlined above.

# Response

**Published:** 2022-05-29

**Authors:** Temidayo S Omolaoye

**Affiliations:** Mohammed Bin Rashid University of Medicine and Health Sciences, United Arab Emirates

**Comment:**

 The manuscript presented to me for review concerns the genetic causes of male infertility in the Middle East and North Africa (MENA) region. The topic discussed is important and still relevant due to its clinical significance and implications. Based on a literature review (different electronic databases were searched), the authors showed that genetic aberrations (Y chromosome microdeletion, deletion or gene mutation, gene polymorphism or copy number variation, chromosomal disorders, mitochondrial mutation) are a common cause of male infertility in the MENA region. The obtained research results are comprehensively presented in Table 1 and described in the text. Taking into account the obtained data, the authors rightly suggest that genetic tests should be part of the diagnosis of male infertility. Undoubtedly, such diagnostics will allow for proper therapeutic management, especially in the case of using ART. Generally, the text is written correctly in terms of its content. However, the manuscript requires revision prior to indexing.

**Response:**

 The authors would like to thank the reviewer for the time and effort expended to review this work and thereby adding to the scientific merit of this study. Suggestions made by the reviewer have been addressed appropriately. 

**Comment:**

 The most important remarks are listed below:

 Abstract: “…30% of these cases remain idiopathic.” This data should be updated. Idiopathic infertility accounts for 30-50% of male infertility (Agarwal et al., 2019,2021).

**Response:**

 The information has been updated to read “about 30-50% of these cases remain idiopathic”.

**Comment:**

 There is no information in the Introduction about idiopathic failure.

**Response:**

 The below sentences have been inserted in the text to address idiopathic infertility, from line 51-56.

 “It has been reported that around 60% of the total cases are attributable to the male factor, of which up to 50% are idiopathic (Agarwal *et al.* 2019, 2021). Unlike unexplained male infertility which sometimes is characterized with normal semen parameters, idiopathic male infertility is diagnosed in the presence of altered semen characteristics without an identifiable cause and the absence of female factor infertility (Hamada *et al.* 2011, Agarwal *et al.* 2019)”.

**Comment:**

 “Y chromone (?) microdeletion”; “…P or Q arm of…” should be p arm or q arm

**Response:**

 Corrected in the text. Line 135.

**Comment:**

 The content on the molecular structure of the Y chromosome should also be presented in the form of a diagram (structure of the Y chromosome with regions and genes marked).

**Response:**

 A new diagram has been inserted in the text as Figure 3

**Comment:**

 The caption under figure 2 should be corrected: ”…the genetic alteration the genetic alteration.”

**Response:**

 The repeated sentence has been deleted.

**Comment:**

 Information on ROS is contained in two sections: “Evidence of genetic mutations in the MENA region” and “Evidence of mitochondria mutation in the MENA region”. I suggest that they be written exhaustively only in the section: “Evidence of mitochondria mutation in the MENA region”. They should be expanded and superseded with the latest literature. The text includes many mental abbreviations.

**Response:**

 We avoided focusing on ROS because several review articles have elaborated on the matter. However, we have cited more studies that discussed to topic in greater detail for further reference.

 The sub-section addressing Evidence of Mitochondria Mutation in the MENA region has been updated. Line 388-401.

**Comment:**

 Section: “CATSPER channel protein”. “Avenarius et al. (2009) carried out a study to report the relationship between variation in the CATSPER1 channel and infertility amongst (???) Iranian men. The study showed that the CATSPER1 channel protein is responsible for the calcium influx during the hyperactivity phase of sperm after ejaculation. Thus, any variation in this protein channel can disrupt the hyperactivity phase, evidently leading to infertility (Avenarius et al. 2009).” This fragment of text should be corrected. Hyperactivation of ejaculated sperm cells (the spermatozoa movement is changed) is the result of their capacitation in the female reproductive system and enables the fertilization process. The authors used too much shortcut. References should be updated.

**Response:**

 This section of the manuscript has been revised. Please see below and line 266-276 in the text.

 The study was able to identify insertion mutations, which led to premature stop codons and consequently variation in the CATSPER 1 protein (Avenarius *et al.* 2009). The CATSPER 1 protein is part of a tetrameric voltage gated calcium channel which are highly conserved in humans and mice.  Carlson et al. showed the necessity of CATSPER 1 for Ca2 ^+^ entry into the flagellum and for Ca2 ^+ ^mediated hyperactivated sperm motility (Carlson *et al.* 2003). Thus, an abnormality in the CATSPER 1 protein may hinder the calcium-mediated sperm functions. Once sperm enters the female reproductive tract, it undergoes the calcium mediated process of capacitation. When capacitation occurs successfully, the sperm is able to carry out its role in fertilization. Thus, it was suggested that variation in the CATSPER 1 channel may hinder the process of capacitation and consequently leading to infertility (Avenarius *et al.* 2009).

**Comment:**

 No development of the abbreviation PiRNAs, TDRD;  “PIWI (comment of reviewer: gene or protein,  italics means gene) and TDRD proteins are…”… HIWI genes… “(???)

**Response:**

 Statement has been revised appropriately.

**Comment:**

 The text should be read very carefully and checked in terms of spelling, style and abbreviations.

**Response:**

 The entire manuscript has been thoroughly proofread.
